# Acoustic Modulation of Individual Nanowire Quantum Dots Integrated into a Hybrid Thin-Film Lithium Niobate Photonic Platform

**DOI:** 10.1021/acs.nanolett.4c03402

**Published:** 2024-09-26

**Authors:** Thomas Descamps, Tanguy Schetelat, Jun Gao, Philip J. Poole, Dan Dalacu, Ali W. Elshaari, Val Zwiller

**Affiliations:** † Department of Applied Physics, KTH Royal Institute of Technology, Roslagstullsbacken 21, Stockholm 10691, Sweden; ‡ National Research Council Canada, Ottawa K1A 0R6, Ontario, Canada; § Single Quantum BV, Delft 2629, The Netherlands

**Keywords:** quantum dots, single-photon source, surface acoustic waves, thin-film lithium niobate, integrated photonics

## Abstract

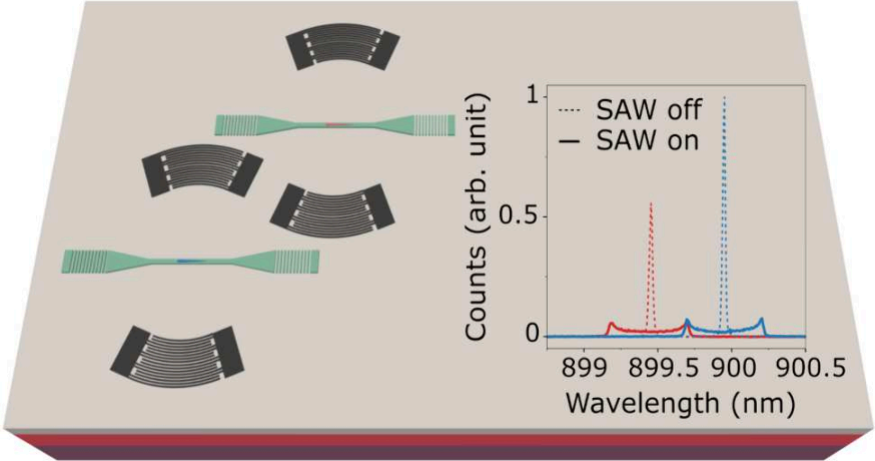

Surface acoustic waves are a powerful tool for controlling quantum systems, including quantum dots (QDs), where the oscillating strain field can modulate the emission wavelengths. We integrate InAsP/InP nanowire QDs onto a thin-film lithium niobate platform and embed them within Si_3_N_4_-loaded waveguides. We achieve a 0.70 nm peak-to-peak wavelength modulation at 13 dBm using a single focused interdigital transducer (FIDT) operating at 400 MHz, and we double this amplitude to 1.4 nm by using two FIDTs as an acoustic cavity. Additionally, we independently modulate two QDs with an initial wavelength difference of 0.5 nm, both integrated on the same chip. We show that their modulated emissions overlap, demonstrating the potential to bring them to a common emission wavelength after spectral filtering. This local strain-tuning represents a significant step toward generating indistinguishable single photons from remote emitters heterogeneously integrated on a single chip, advancing on-chip quantum information processing with multiple QDs.

Surface acoustic waves (SAWs), with their capacity to interact mechanically with both the supporting crystal and the materials on its surface, have shown significant interest for controlling various quantum systems, including superconducting qubits,^1−3^ spin qubits,^4−6^ quantum optomechanical cavities,^7,8^ and single-photon emitters based on defect centers^9−11^ or III/V semiconductor quantum dots (QDs). In the latter case, the oscillating electric field created by the SAW propagating on a piezoelectric medium was used to transport charge carriers to the QD and to control the emitter’s charge state.^12−14^ Additionally, the oscillating strain field induced by the SAW modulates the energy levels of the QD.^15,16^ Utilizing this property, coherent coupling between acoustic phonons and single photons^17−20^ as well as single-photon frequency shifting have been demonstrated.^21−23^ These investigations, predominantly focused on a single QD, could be extended to multiple emitters on the same chip, each independently modulated by a SAW to tune their emission wavelengths. This advancement would be of technological interest, as it would address the variance in emission wavelengths of these sources,^24−27^ a major limitation for their applications in integrated linear quantum computing^28−31^ and quantum communication protocols based on quantum interference effects,^32,33^ where photon indistinguishability is paramount. Typically generated by driving an interdigital transducer (IDT) patterned on a piezoelectric substrate with a microwave signal, SAWs offer several advantages over other tuning mechanisms. First, the emission wavelength can be either red-shifted or blue-shifted, unlike thermo-optic schemes based on local heating of the source, which always result in a red-shift.^34,35^ Second, QDs can be directly modulated without the need for doping the heterostructure and making electrical contacts, as required for Stark-effect-based tuning.^36,37^ Lastly, the localized strain field and fabrication simplicity of this method make it more scalable compared to other strain mechanisms, such as those using global static fields applied with piezoelectric substrates^38,39^ or MEMS technologies employing suspended films.^40,41^ In this work, we examine InAsP/InP nanowire (NW) quantum dots (NWQDs), which are known for being bright sources of indistinguishable single photons with a low multiphoton emission probability.^27,42,43^ Unlike the monolithic approach, where self-assembled QDs are embedded in waveguides etched into the III/V heterostructure,^31,44^ the site-controlled NWs are picked up and placed^45,46^ onto an unreleased thin-film lithium niobate (LN) platform, as this strong piezoelectric material enables more efficient electro-mechanical transduction. The NWs are then integrated into Si_3_N_4_-loaded waveguides^47−49^ and positioned at the center of an acoustic delay line consisting of focused interdigital transducers (FIDTs). We achieve a modulation of the emission wavelength with a peak-to-peak amplitude of 0.70 nm by driving a single FIDT at 400 MHz with a microwave power of 13 dBm, and this modulation amplitude is twice as large by driving two FIDTs as the acoustic cavity. Finally, we demonstrate that two waveguide-integrated NWQDs, whose emission wavelengths differ by 0.5 nm, can be brought to a common emission wavelength by using SAWs. This result paves the way for generating indistinguishable single photons from multiple remote QDs heterogeneously integrated on a single photonic chip.

An optical microscope image of the hybrid quantum photonic platform developed in this work is shown in Figure 1a, featuring four nanowire quantum dots, each integrated into a photonic waveguide and positioned within an acoustic delay line. The wurtzite InP NWs embedding individual InAsP QDs^50,51^ emitting around 900 nm were picked up with a nanomanipulator inside a scanning-electron microscope (SEM) and transferred to a 300 nm thick Y-cut thin-film LN chip with 4.7 μm buried SiO_2_. The NWs were oriented along the crystallographic *Z*-axis. A 350 nm thick Si_3_N_4_ loading layer was then deposited by plasma-enhanced chemical vapor deposition (PECVD) on the whole surface and etched to define the photonic elements. Since Si_3_N_4_ has a slightly lower refractive index than that of LN, the optical mode is spatially confined beneath the Si_3_N_4_ strip.^47−49^ For the 1.2 μm wide fabricated waveguide, the spatial distribution of the mode is presented in Figure 1c (cross section BB). We estimate that 80.1% of the total optical mode intensity is confined within the LN slab and 18.6% in the Si_3_N_4_ strip, and the remainder either leaks into the buried SiO_2_ or escapes from the structure. The waveguides are terminated by grating couplers with a simulated efficiency of 45%, which were used for exciting the QD and collecting the emitted photons. An SEM image of the photonic channel around the NW is presented in Figure 1b, while an SEM image of the waveguide-integrated NW is shown in Figure 1c. The alignment of the waveguide relative to the NW was well-achieved, with a 150 nm large gap present between them, as Si_3_N_4_ did not reproducibly adhere to the InP during deposition. The tapered shape of the NW favors an adiabatic mode transfer of the transverse electric (TE) mode of the NW to the fundamental TE mode of the waveguide. Finite-difference time-domain simulations (Lumerical) were conducted assuming lossless materials and yielded a coupling efficiency of 78%. Two of the four NWs, hereafter referred to as NW1 and NW2 (with quantum dots QD1 and QD2, respectively), were selected post-encapsulation based on their emission properties to be at the center of two acoustic delay lines. Each delay line comprised two opposing FIDTs made of chromium with a common geometric focal point. Both FIDTs feature the same geometry, with a period of Λ = 10 μm repeated *N* = 20 times, a 400 μm focal length, and a 45° opening angle. By orienting the transducers toward the *X*-axis of the crystal, a shear SAW with a fundamental frequency at ν_0_ = 402.4 MHz can be excited. The displacement profile of this SH0 mode is shown in Figure 1d. Based on the delta-function model,^52^ the frequency response of the IDT is well approximated around the resonance frequency by sinc(*N*π(ν/ν_0_ – 1)). With our design parameters, the half-power bandwidth of the central lobe is calculated to be Δν = 17.8 MHz. The in-plane displacement is perpendicular to the SAW propagation direction and is mostly confined to the LN and SiO_2_ layers. The wave velocity is *c*_SH0_ = Λ × ν_0_ = 4024 m/s. The primary component of the associated strain tensor is shear element ε_*zx*_, whose profile is represented in Figure 1e. The presence of nonzero strain at the center of the nanowire, positioned on top of the thin-film LN, indicates that the QD experiences an oscillating strain field as the SAW propagates. Compared to a straight-electrode IDT, which generates plane-wave SAWs, a focused IDT, whose electrodes are shaped as arcs of periodically spaced concentric circles, can be used to enhance the SAW intensity. The SAW radiated by the fabricated FIDT was simulated with COMSOL, and its transverse displacement field is displayed in Figure 1f. The maximum acoustic amplitude is reached at *x*_0_ = 470 μm, offset by 70 μm from the geometric focal point of the FIDT. The acoustic intensity can be fitted to a Gaussian beam profile to extract a Rayleigh length of *x*_R_ = 60 μm. Compared to a straight-electrode IDT, the acoustic field at the beamwaist is increased by a factor of 4.1, and at the geometric focal point by a factor of 2.7 (Supporting Information, Section S5). The strain field experienced by the QD is therefore significantly enhanced due to the focusing capability of the FIDT.

**Figure 1:**
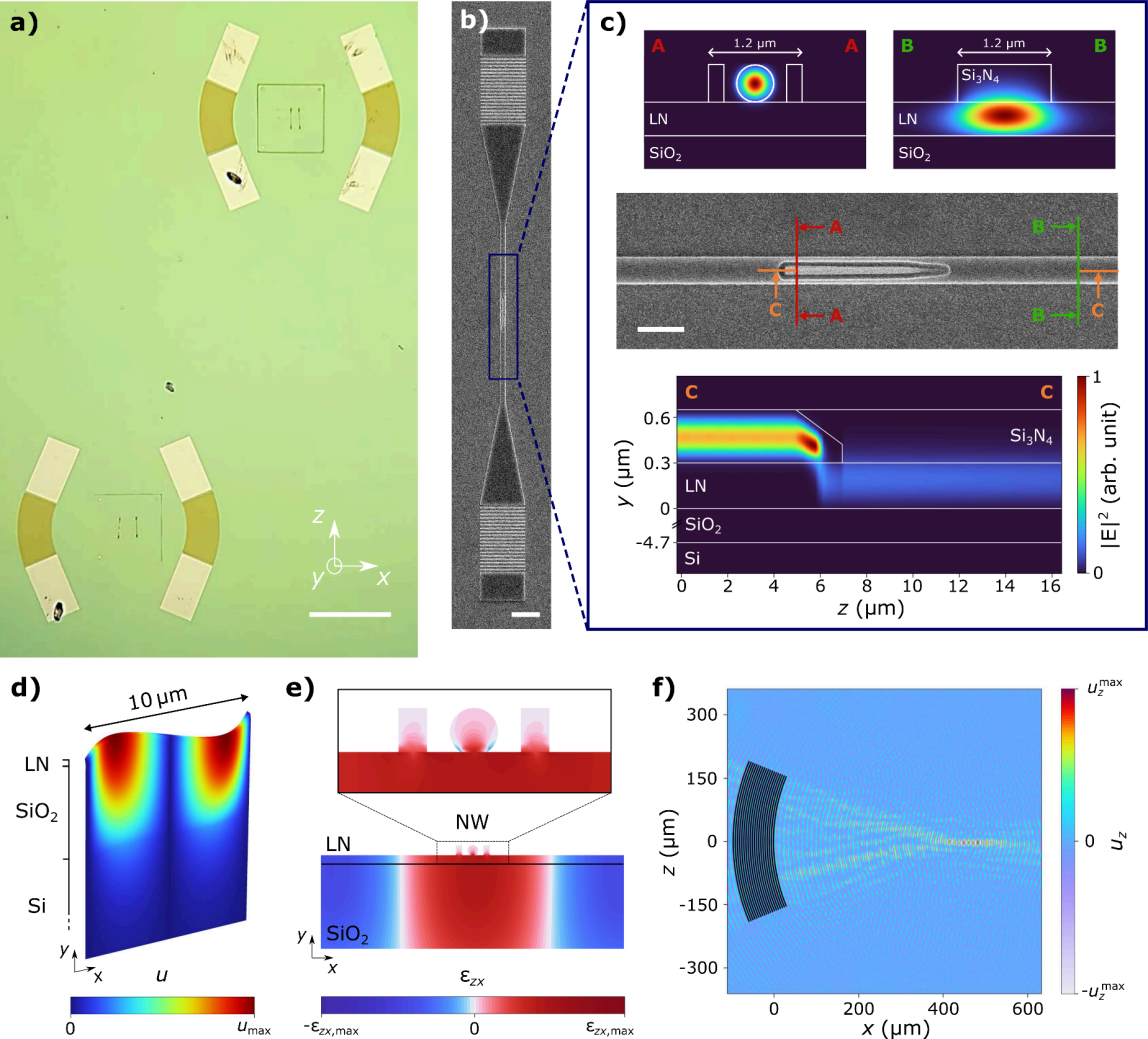
(a) Optical microscopy image of two acoustic delay lines designed for independent acoustic modulation. Within each delay line, two nanowires are integrated into their own Si_3_N_4_-loaded thin-film LN waveguides. The NW at the center of the top (bottom) acoustic cavity is labeled NW1 (NW2). The scale bar is 500 μm. (b) Scanning electron microscope image of a nanowire inside a waveguide, taken from a similar device. The grating at the bottom is used for coupling photons from the waveguide to out-of-plane and vice versa. The scale bar is 5 μm. (c) Scanning electron microscope image of the integrated nanowire. The scale bar is 2 μm. Cross section AA shows the fundamental mode confined in the NW, while cross section BB displays the fundamental TE mode of the Si_3_N_4_-loaded thin-film LN waveguide after the mode transfer region. Cross section CC illustrates the optical TE mode transfer from the NW to the strip-loaded waveguide. The refractive indices used in the simulation are *n*_Si_3_N_4__ = 2.02, *n*_LN_o__ = 2.25, *n*_LN_e__ = 2.17, and *n*_SiO_2__ = 1.45. (d) Displacement profile of the shear SAW mode SH0 obtained by a COMSOL simulation. (e) Strain profile generated by the SAW in the upper layers of the sample and in the NW placed on top. (f) Displacement field of a SAW excited at 400 MHz by a FIDT with a 400 μm focal length and a 45° opening angle. The FIDT has a period of 10 μm with two electrodes per period.

The sample was investigated at 1.8 K in a dry cryostat configured for confocal micro-photoluminescence (PL) measurements and equipped with high-frequency cables. An 80 MHz pulsed-laser (measured 80.026 MHz) was focused with a microscope objective on one grating coupler to excite the waveguide-integrated NWQD above-band at 800 nm. Due to the limited field of view of the microscope, the PL signal propagating toward the same grating coupler was collected by the same microscope objective, dispersed by a 750 cm focal length spectrometer, and detected by a liquid-nitrogen-cooled charge-coupled device (CCD) camera. A two-channel analogue signal generator was used to apply sinusoidal radio frequency (RF) signals with adjustable power *P*_RF_ and phase difference Δϕ to one or both FIDTs of the delay line.

Figure 2a displays the PL spectrum of QD1 without acoustic modulation at an excitation power of 500 nW. In the following, we investigated the brightest line at 899.46 nm, which we attribute to the charged exciton based on count rate power dependence measurements (Section S2) and previous studies on this type of nanowire QD.^43,53^ After being filtered with a monochromator (0.1 nm bandwidth), the purity of the single-photon source was assessed in a Hanbury Brown–Twiss measurement (inset of Figure 2a). The signal was detected by superconducting nanowire single-photon detectors and counted by a time tagging device. The second-order correlation function was fitted with a sequence of equidistant photon pulses assuming a monoexponential decay, yielding a radiative decay time of τ = 0.88 ± 0.02 ns. The suppression of the peak at zero time delay indicates single-photon emission, and the ratio of the area of the zero time delay peak to the area of the finite time delay pulses gives *g*^(2)^(0) = 0.010 ± 0.002. When a 400 MHz RF signal is applied to the FIDT, the sinusoidal modulation of the strain field around the QD induces a modulation of its bandgap energy at the same frequency, causing the spectral lines to oscillate around their unstrained energies.^21^ This driving frequency remains within the bandwidth of the IDT without a major loss of performance. Spectral broadening already becomes noticeable for all peaks at approximately *P*_RF_ = −10 dBm and reaches 0.70 nm peak-to-peak at 13 dBm (Figure 2b). This optomechanical coupling arises exclusively from shear strain modulating the energy levels of the QD, an effect less commonly studied compared to normal strain coupling. The optical modulation occurring at the SAW frequency indicates a good mechanical contact between the lithium niobate thin film and the nanowire despite the absence of encapsulation. The contact does not seem to deteriorate even at moderate RF powers, as evidenced by a stable increase in the modulation. The broadening also remains symmetric around the unstrained emission, indicating that heating of the QD is effectively mitigated at moderate RF powers.^44^ By choosing a modulation frequency lower than the decay rate of the emitter, we avoid phonon sidebands around the central emission line. Then, both FIDTs forming the delay line are driven at 400 MHz with two independent microwave channels to produce two counter-propagating SAWs whose superposition forms a standing wave. A minor performance discrepancy between the two FIDTs, attributed to fabrication imperfections, is compensated by applying slightly less power to the first FIDT (*P*_RF,1_ = 12.5 dBm) compared to the second (*P*_RF,2_ = 13 dBm). The standing wave generates a pattern of nodes (points of zero displacement) and antinodes (points of maximum displacement) whose position with respect to the nanowire can be adjusted by modifying the phase difference Δϕ of the two RF signals. Figure 2c illustrates the modulation of the brightest emission line of QD1 as a function of Δϕ. When both signals are in phase, the nanowire lies at an antinode of the standing wave, resulting in a modulation amplitude that is twice that obtained with a single propagating SAW. Conversely, the acoustic modulation is completely suppressed when a π phase shift is imposed between the two FIDTs. The dynamic spectral broadening 2Δ*E* was extracted from the data by fitting it to a time-integrated oscillating Lorentzian emission line.^15^ In Figure 2d, 2Δ*E* is plotted as a function of Δϕ. Its trend follows the theoretical expression 2Δ*E* = 2Δ*E*_0_ × 2|cos((Δϕ + γ)/2)|, where 2Δ*E*_0_ is the energy broadening when only one of the FIDTs is excited. The fitting parameter γ = −2.0° represents a residual phase shift attributed to a slight length mismatch of the RF cables within the cryostat. The good fitting also confirms that heating has no noticeable effect, even when both FIDTs are driven simultaneously on the same chip.

**Figure 2:**
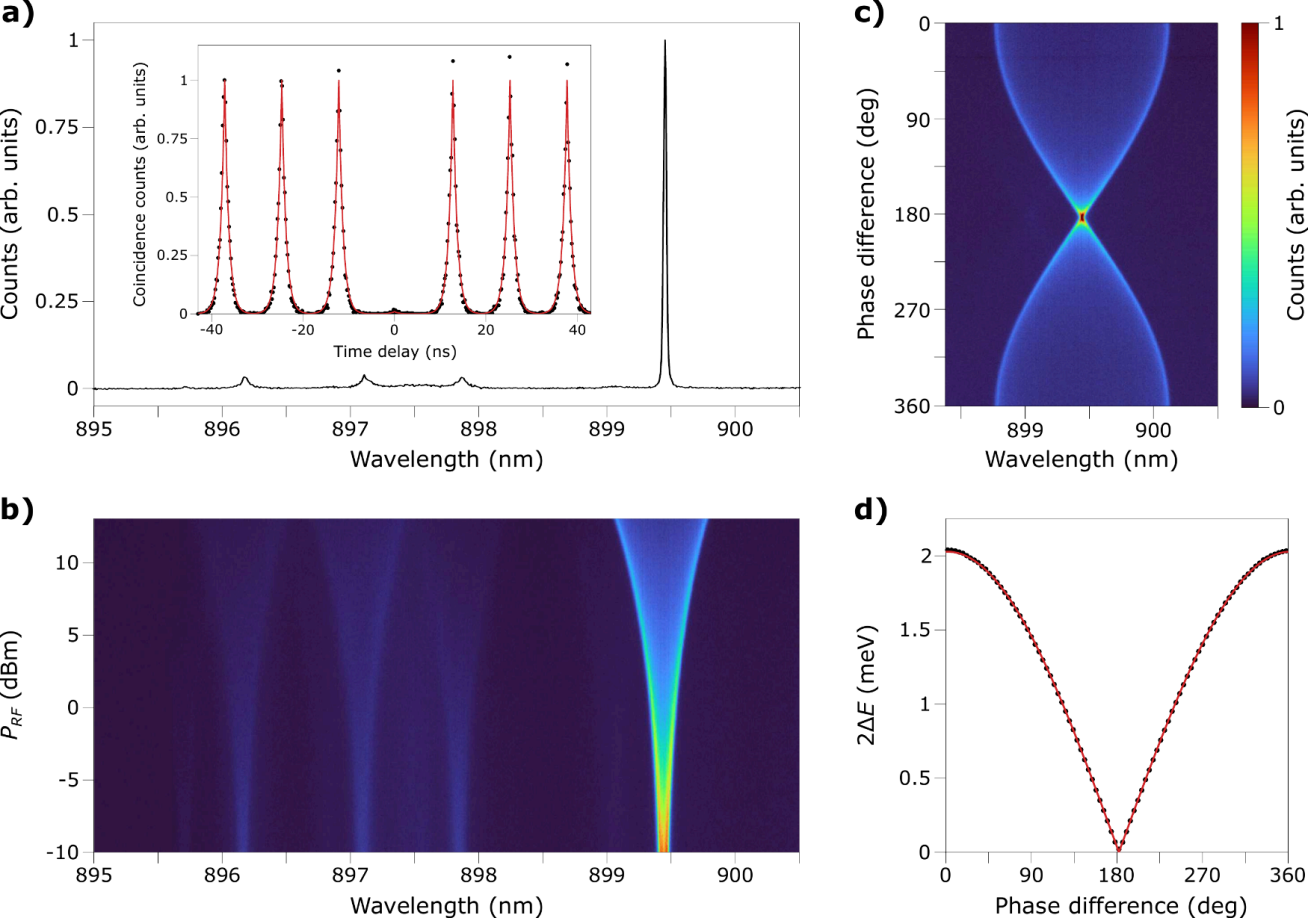
(a) PL spectrum of QD1 without modulation. Inset, second order correlation function of the brightest emission peak. The fitting function is detailed in the main text. (b) Measured optical modulation induced by a single SAW as a function of *P*_RF_ (generation at 400 MHz). The colorbar is the same as that of (c). (c) Optical modulation of the brightest emission peak by two counter-propagating SAWs as a function of their relative phase Δϕ. For this measurement, both FIDTs are excited at 400 MHz (*P*_RF_ = 12.5 dBm). (d) Strain-induced energy splitting as a function of Δϕ. The fitting function in red is detailed in the main text. For all of the measurements, the QD was excited with a 80 MHz pulsed-laser at 500 nW.

Similarly to QD1, the modulation performance of QD2 in the second delay line was investigated. For both QDs, the spectral broadening is plotted as a function of the driving RF power on a logarithmic scale (Figure 3a). Over the studied power range, the modulation of NW2 is on average 21% smaller than that of NW1. This discrepancy is attributed to variations in the performance of the FIDTs, and to different adhesions of the NWs on the lithium niobate. In both cases, the strain-induced broadenings follow the power law 2Δ*E* ∝ (*P*_RF_)^α^, where α = 0.489 ± 0.001 for NW1 and α = 0.474 ± 0.002 for NW2. These coefficients closely approach the ideal value of α = 0.5 expected for deformation potential coupling, indicating that the observed broadening primarily arises from optomechanical coupling.^15^ As shown in Figure 3b, the wavelength of the charged exciton line of NW2 is 0.5 nm greater than that of NW1. These different emission wavelengths can stem from multiple factors, from the growth process^27^ to the static strain and charge environment after transfer to the host substrate. Two separate RF signals at 400 MHz were employed to excite one FIDT from each delay line in order to modulate both QDs independently. A higher power was applied to the FIDT of the second delay line to obtain identical spectral broadenings for both nanowires. Once per acoustic cycle, the two QDs emitted at a common wavelength of 899.70 nm.

**Figure 3:**
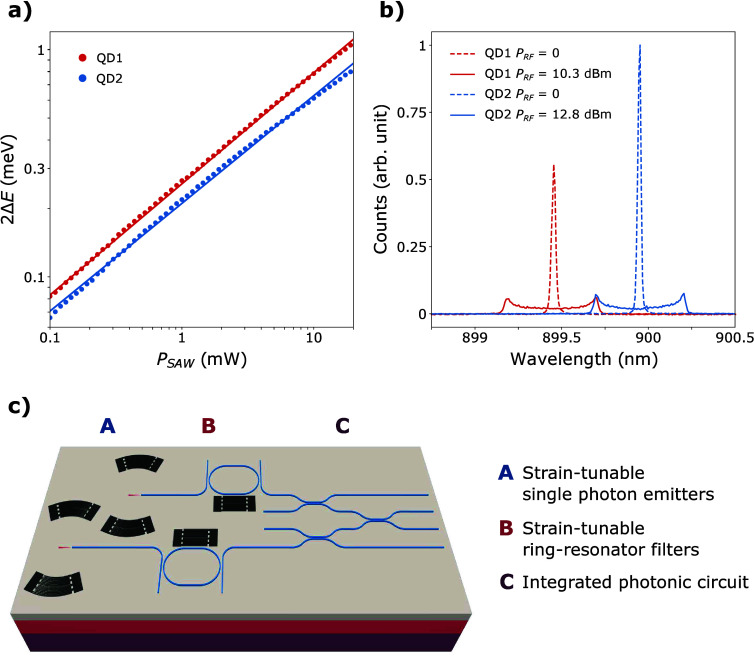
(a) Strain-induced spectral broadening of the charged exciton line of quantum dots 1 and 2 as a function of *P*_RF_. The solid lines are linear fits. (b) Emission peaks of QD1 and QD2 without (dashed lines) and with (solid lines) SAW-induced modulation. For all of the measurements, the QDs were excited with an 80 MHz laser at 500 nW. (c) Artistic image of two strain-tunable NWQDs integrated in a hybrid thin-film LN photonic platform, which comprises strain-tunable ring-resonator filters injecting resonant photons into an integrated photonic circuit.

To extract the modulated photons from both QDs at this common wavelength, synchronized microwave sources with a π-shift between them are required, ensuring that one photon is blue-shifted while the other is red-shifted. Depending on the ratio of the exciton lifetime to the SAW period, two different processing schemes can be considered. If the SAW period is longer than the radiative decay time, the strain field and the resulting deformation potential around the QD can be considered quasi-static. Having the SAW frequency be an integer multiple of the repetition rate of the pulsed lasers allows for repeated optical excitation of the QD at a fixed point in the acoustic cycle. As a result, the emission would consistently fall within a desired energy range, eliminating the need for spectral filtering. Conversely, if the strain field varies during the exciton recombination time, different emission wavelengths arise and post-emission filtering becomes necessary to ensure spectral overlap. This can be achieved by integrating a photonic resonator after each nanowire QD, as illustrated in Figure 3c. These resonators can be tuned to the common emission wavelength using electro-optic schemes^49,54^ or SAWs,^55−57^ provided a tunable phase delay to compensate for propagation delay. In addition, a free spectral range larger than the peak-to-peak modulation amplitude of the QDs ensures that only photons at the common wavelength are transmitted through the drop-port to the integrated photon circuit for further processing.

A statistical analysis of similar NWQDs, emitting at slightly longer wavelengths than those investigated here, revealed a Gaussian distribution of the emission wavelengths with a standard deviation of 4.65 nm.^27^ Although the measurement presented above demonstrated that two selected NWQDs could be tuned in resonance, achieving a larger spectral modulation would relax the selection process. One straightforward improvement would be to increase the driving RF power beyond 13 dBm, provided that sample heating does not deteriorate spectral tuning.^44^ By extrapolating the power law observed in Figure 3a, we estimate that a dynamic broadening of 1.16 nm can be reached at a microwave power of 17.1 dBm with a single FIDT, potentially bringing 10% of such NWQDs to a common wavelength once per acoustic cycle. To reduce ohmic losses, a lower resistivity metal such as aluminum, gold, or platinum^58^ could be used instead of chromium for the FIDT electrodes. Placing the QD at an antinode of a standing-wave created by driving both FIDTs of the delay line is another effective approach to improve modulation performance by a factor of 2, as demonstrated in Figure 2d. A similar effect can be obtained by positioning the QD between two SAW mirrors and exciting the acoustic cavity with only one IDT,^59^ thereby reducing the thermal load by half. Furthermore, the SH0 mode profile (Figure 1d) shows that the SAW is confined in both the LN and silica layers, hence reducing the acoustic energy at the surface. Higher mechanical confinement, and thus enhanced optomechanical modulation, could be achieved by releasing the LN,^22^ although this would involve a more challenging fabrication process and result in a more fragile device. While increasing the strain modulation amplitude could bring a greater fraction of QDs to a common wavelength, this improvement comes at the cost of a reduced count rate after spectral filtering. For instance, to overcome the 0.5 nm wavelength difference between QD1 and QD2, the count rate would decrease to about 23% after spectral filtering with the monochromator (Section S4). Our strain-modulation scheme can also be scaled to more than two emitters on the same chip, without additional fabrication complexity. In this regard, the footprint of the FIDT can be shrunk from a focal length of 400 to 100 μm with a slight reduction of the maximum transverse displacement of the SAW by 15% (Section S5). Finally, obtaining photons at the same wavelength from remote emitters is a necessary but insufficient condition for ensuring a high degree of indistinguishability. Each source must ideally emit Fourier transform-limited photons with matching radiative rates.^31,60^ Under above-band excitation, the exciton lines from QD1 and QD2 exhibit similar lifetimes (0.88 and 0.85 ns, respectively) and comparable line widths (8.34 and 6.07 GHz, respectively), as detailed in Section S6. This uniformity, resulting from the optimized growth process, combined with the SAW-based wavelength-tuning approach, highlights the potential of nanowire QDs as integrated sources of indistinguishable photons.

In summary, we successfully transferred InAsP/InP nanowire quantum dots on a thin-film lithium niobate platform and heterogeneously integrated them into hybrid photonic waveguides through Si_3_N_4_ strip loading. By operating a single focused interdigital transducer at 400 MHz, we excited and coupled a shear SAW to the energy levels of a QD, resulting in a modulation of the emission wavelength by 0.70 nm at 13 dBm. By driving both FIDTs of the delay line, we could either double this modulation or suppress it altogether, depending on the phase difference between the driving RF signals. This local strain tuning approach allowed us to bring two waveguide-integrated NWQDs with a 0.5 nm wavelength to a common wavelength once per acoustic cycle. This represents a crucial step toward generating indistinguishable single photons from multiple remote emitters on a single photonic chip. Photons brought into resonance can then be filtered using resonators operating at the same frequency as the FIDTs and subsequently manipulated with photonic circuits for integrated quantum photonic applications.

## Supporting Information Available

Device fabrication; count rate power dependence; photoluminescence spectrum of QD2; postemission filtering; FIDT acoustic field simulations; and lifetimes and line widths (PDF)
