# Supplementary material for: Acoustic Modulation of Individual Nanowire Quantum Dots Integrated into a Hybrid Thin-Film Lithium Niobate Photonic Platform
Source: Nano Lett. 2024 Sep 26;24(40):12493–500. doi: 10.1021/acs.nanolett.4c03402 (PMC11468731; doi:10.1021/acs.nanolett.4c03402)
Supplement: Supplementary file 1 — nl4c03402_si_001.pdf [file nl4c03402_si_001.pdf]

SUPPLEMENTARY
Acoustic modulation of individual nanowire quantum dots integrated into a hybrid thin-film lithium niobate photonic platform

Thomas Descamps,[1,2,*] Tanguy Schetelat,[1,2] Jun Gao,[1] Philip J. Poole,[3] Dan Dalacu,[3] Ali W. Elshaari,[1] and Val Zwiller[1,4,†]

[1]*Department of Applied Physics, KTH Royal Institute of Technology, Roslagstullsbacken 21, 10691 Stockholm, Sweden*

[2]*These authors contributed equally to this work.*

[3]*National Research Council of Canada, Ottawa, Ontario K1A 0R6, Canada*

[4]*Single Quantum BV, Delft, The Netherlands*

CONTENTS

S1. Device fabrication 2

S2. Count rate power dependence 3

S3. Photoluminescence spectrum of QD2 4

S4. Post-emission filtering 5

S5. FIDT acoustic field simulations 7

S6. Lifetimes and linewidths 9

References 9

* descamps@kth.se

† zwiller@kth.se

S1. DEVICE FABRICATION

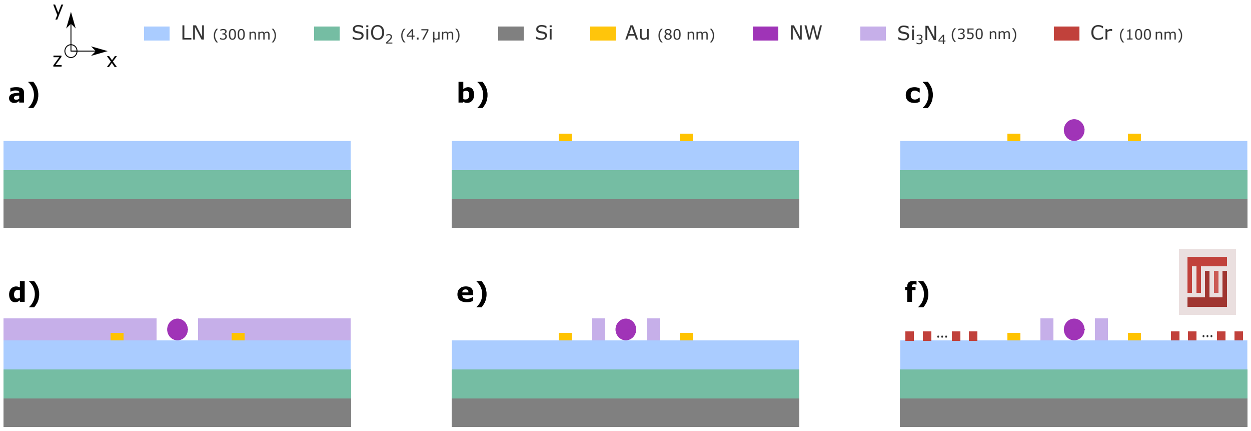

Fig. S1. Fabrication workflow.

The LNOI surface (Fig. S1(a)) was coated with positive resist (AR-P 6200.9), and alignment markers were patterned using electron-beam lithography (EBL). After development, a Ti/Au layer was evaporated and subsequently lifted-off (Fig. S1(b)). The nanowires were transferred from the growth substrate to the chip (Fig. S1(c)) using nano-manipulators mounted inside a scanning electron microscope (SEM). A 350 nm-thick Si_3N_4 loading layer was then deposited at 300 °C using plasma-enhanced chemical vapor deposition (PECVD) on the entire surface (Fig. S1(d)). This process was carried out at 1000 mTorr with a gas mixture of 350 sccm 5 %-diluted SiH_4 and 20 sccm NH_3. The deposition involved repeated cycles of high-frequency plasma (13.56 MHz - 50 W) and low-frequency plasma (100 kHz - 50 W) for 12 s and 8 s, respectively. The surface was then coated with negative EBL resit (ma-N 2403) and the photonic elements were patterned by EBL according to the positions of the nanowires. The pattern was transferred to the Si_3N_4 by reactive ion etching in a CHF_3/SF_6 plasma to define the photonic elements (Fig. S1(e)). The waveguides were 1.2 µm-wide and the grating couplers had a period of 590 nm with a filling factor of 0.5. Finally, the FIDTs were created by EBL followed by chromium evaporation and lift-off (Fig. S1(f)). The FIDT had a split-52 design (period showed in inset of Fig. S1(f)) with an electrode width of 1 µm, allowing for SAW excitation at a fundamental frequency of $f_1 = 402.4$ MHz and harmonics $f_n = nf_1$ for $n = 2, 3$, and 4.

S2. COUNT RATE POWER DEPENDENCE

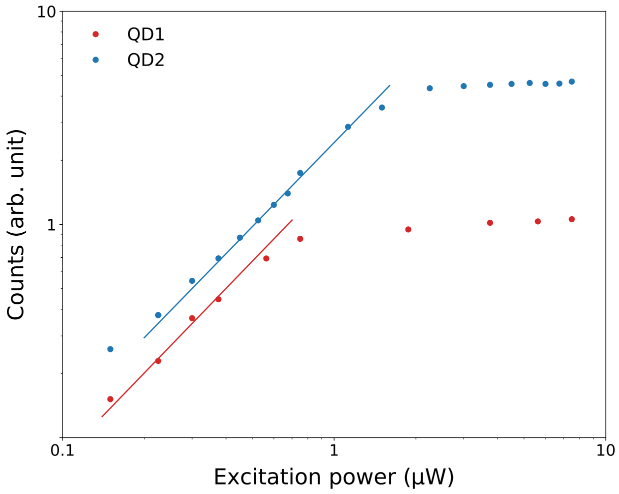

Fig. S2. Count rate power dependence of the emission lines investigated for QD1 and QD2 (logarithmic scale). The straight lines are linear fit.

The count rate power dependence of the emission lines investigated in the main text for QD1 and QD2 is shown in Fig. S2. Before saturation, linear fits to the data show that the count rates increase with exponents 1.32 (QD1) and 1.31 (QD2), consistent with the behaviour of a charged exciton [1]. This identification is further supported by previous studies on similar nanowire QDs, which demonstrated that the lowest energy peak corresponds to the negatively charged exciton [2, 3].

S3. PHOTOLUMINESCENCE SPECTRUM OF QD2

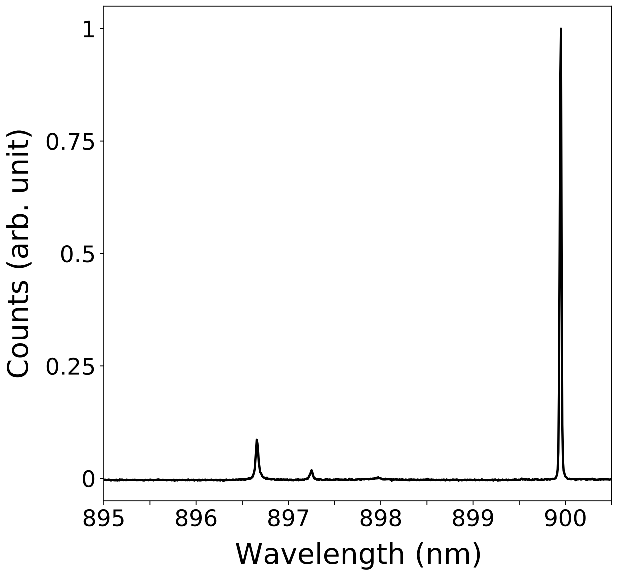

Fig. S3. PL spectrum of QD2 without modulation. The QD was excited with a 80 MHz laser at 500 nW.

S4. POST-EMISSION FILTERING

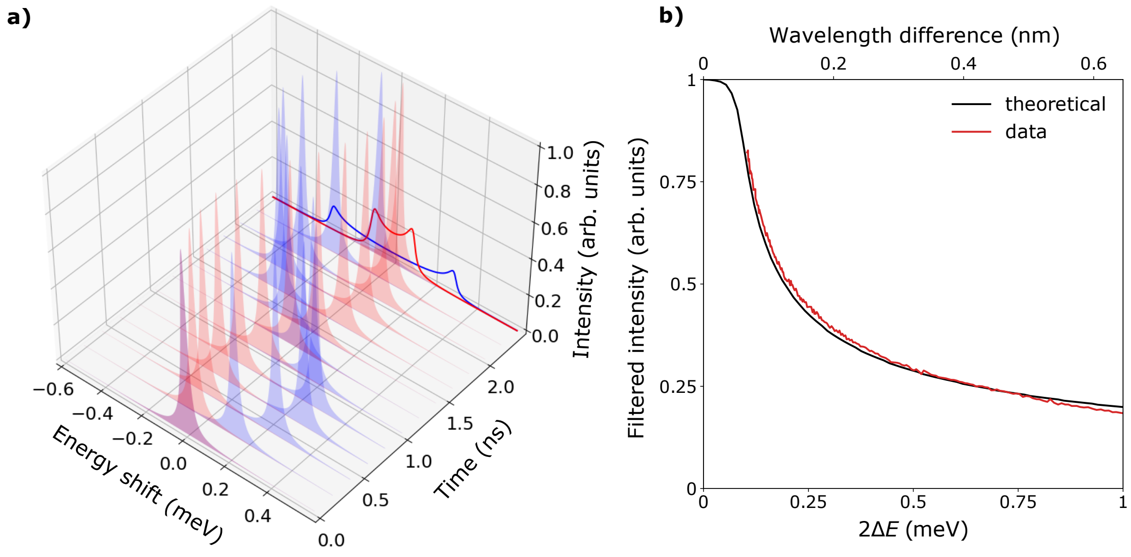

Fig. S4. **a)** Illustration of an oscillating Lorentzian lineshape over one acoustic cycle at two different modulation amplitudes ΔE (0.1 meV and 0.3 meV for the red and blue oscillations, respectively). The time average of these oscillations is shown in the plane at 2.5 ns. **b)** Fraction of the emission intensity filtered by a 0.1 nm bandwidth monochromator as a function of the dynamic spectral broadening $2\Delta E$. The filtering is applied at one of the two maxima of the time-averaged modulated line. The black curve was computed assuming an oscillating Lorentzian lineshape with the same linewidth (34.5 µeV) as the one investigated for QD1. The red curve represents experimental values measured for QD1. The top axis represents the initial wavelength difference between two QDs emitting around 900 nm, which can be compensated by the corresponding modulation amplitude.

Spectral filtering of the modulated emission will inevitably reduce the single-photon count rate. Assuming an oscillating Lorentzian lineshape (linewidth γ), the time-averaged spectrum is given by

$$I(E) \propto \frac{2}{\pi T_{\mathrm{SAW}}} \int_0^{T_{\mathrm{SAW}}} \frac{\gamma}{4\big[E - \Delta E \sin(2\pi t/T_{\mathrm{SAW}})\big]^2 + \gamma^2} \mathrm{d}t \tag{1}$$

where T_{SAW} is the SAW period, and $2\Delta E$ the dynamic spectral broadening. This lineshape broadens with increased ΔE, as illustrated in Fig. S4(a), leading to a decrease in its maximum intensity. If a 0.1 nm filtering window is centered around one of the two maxima,

approximately 38 % of the initial count rates will be retained for a modulation sufficient to overlap two QDs with an initial wavelength difference of 0.2 nm. For a wavelength difference of 0.5 nm, this fraction decreases to about 23 %.

S5. FIDT ACOUSTIC FIELD SIMULATIONS

The acoustic field generated by the FIDT is simulated using COMSOL. Twenty pairs of chromium electrodes, shaped as arcs of concentric circles, were placed on top of a 300 nm-thick Y-cut thin-film LN chip with 4.7 µm buried oxide. The FIDT had a period of 10 µm with two electrodes per period, a 400 µm focal length and a 45 ° opening angle. An oscillating electric potential at 400 MHz was applied to every other electrode while the remaining electrodes were grounded. Perfectly matched layer conditions were imposed on the lateral boundaries of the domain, and the bottom boundary was fixed. The orientation of the axes is the same as shown in Fig. 1 in the main text.

Fig. S5 shows the transverse displacement u_z on the top surface along the direction of SAW propagation at a constant $z = 0$ µm. The envelope of the mechanical oscillations can be fitted to a Gaussian beam profile along its center axis

$$\tilde{u}_z(x) \propto \frac{1}{\sqrt{1 + \left((x - x_0)/x_{\mathrm{R}}\right)^2}}$$

where x_0 is the position of the beam waist, and x_{R} is the Rayleigh length. The fitting parameters are $x_{\mathrm{R}} = 60$ µm and $x_0 = 470$ µm, indicating that the beam's focus is offset from the geometric focus by 70 µm. A similar simulation was conducted for a straight-electrode IDT with an identical period. In this case, the mechanical oscillations exhibit a nearly constant amplitude over the simulated propagation distance. This amplitude is extracted by fitting the data to a simple sinusoidal function, serving as a baseline to evaluate the performance gain of the FIDT. Compared to the IDT, the FIDT generates an acoustic field at the beam waist that is greater by a factor of 4.1, and at the geometric focal point by a factor of 2.7.

The transverse displacement generated by a smaller FIDT with 100 µm focal length but with the same 45 ° opening angle is shown in Fig. S6(a). The SAW is also focused, and the maximum displacement occurs at 15 µm to the geometric focus. The reduction of the footprint of the FIDT is particularly interesting for increasing the density of modulated sources on the same chip. By fitting the envelope of the displacement around the beam waist, we found that the maximum displacement generated by the 100 µm focal length FIDT is slightly reduced by 15 % compared to the 400 µm focal length FIDT (Fig. S6(b)).

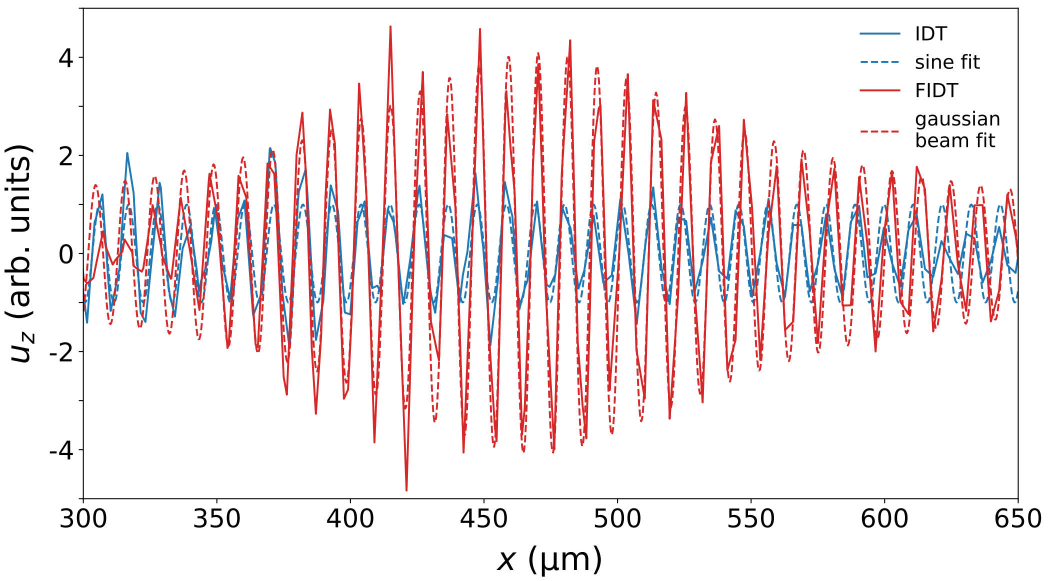

Fig. S5. Simulated transverse displacements u_z of a shear SAW propagating along the X axis of a Y-cut thin-film lithium niobate. The SAW was excited at 400 MHz by an IDT (blue) and an FIDT (red) with similar geometries. The data (solid lines) was fitted (dashed lines) to a sine function for the IDT, and to a Gaussian profile for the FIDT.

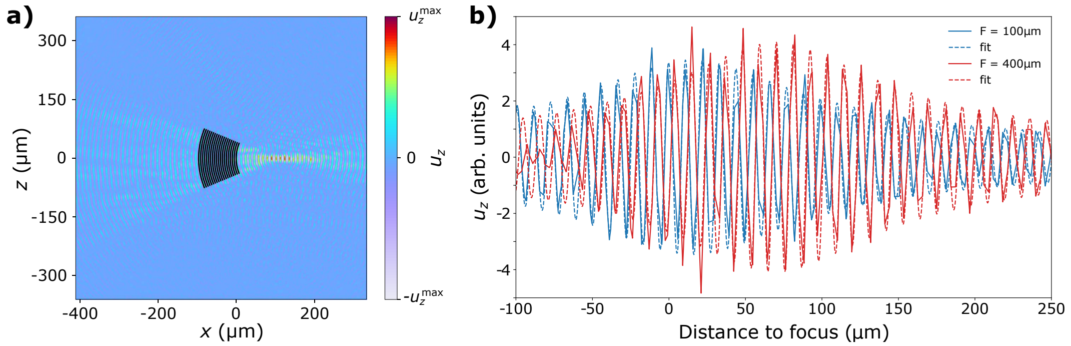

Fig. S6. **a)** Displacement field of a SAW excited at 400 MHz by an FIDT with a 100 μm focal length and a 45 ° opening angle. The FIDT has a period of 10 μm with two electrodes per period. **b)** Simulated transverse displacements u_z of a shear SAW propagating along the X axis of a Y-cut thin-film lithium niobate for two FIDTs with focal lengths F of 400 μm (red) and 100 μm (blue). The data (solid lines) was fitted (dashed lines) to a Gaussian profiles.

S6. LIFETIMES AND LINEWIDTHS

Lifetime measurements were performed on the charged exciton emission lines of QD1 and QD2, yielding similar lifetimes of 0.88 ns and 0.85 ns, respectively. In addition, the linewidths of these emission lines were measured without modulation using a spectrometer with a 7.5 pm resolution (2.8 GHz at 900 nm). Fitting the data to Lorentzian line shapes gives a linewidth of 34.5 µeV (8.34 GHz) for QD1, and 25.1 µeV (6.07 GHz) for QD2. Each peak was then normalized and an overlap of 80 % was obtained.

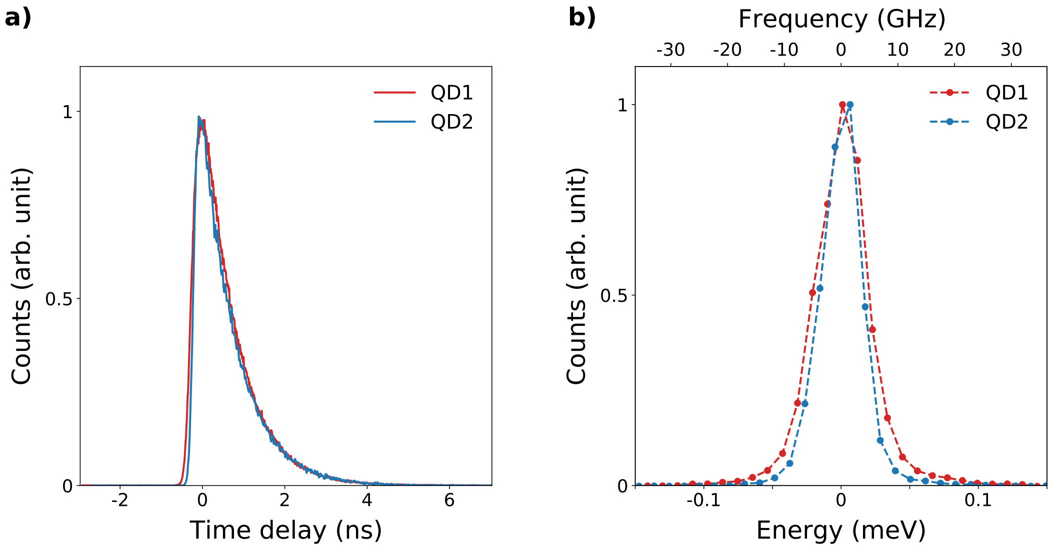

Fig. S7. **a)** Lifetime measurement of the lines investigated for QD1 and QD2. **b)** Spectral linewidths of the lines of both QDs. For both measurements, the QDs were excited with a 80 MHz laser at 500 nW.

[1] M. Weiß, F. J. Schülein, J. B. Kinzel, M. Heigl, D. Rudolph, M. Bichler, G. Abstreiter, J. J. Finley, A. Wixforth, G. Koblmüller, and H. J. Krenner, Radio frequency occupancy state control of a single nanowire quantum dot, Journal of Physics D: Applied Physics **47**, 394011 (2014).

[2] P. Laferrière, E. Yeung, M. Korkusinski, P. J. Poole, R. L. Williams, D. Dalacu, J. Manalo, M. Cygorek, A. Altintas, and P. Hawrylak, Systematic study of the emission spectra of nanowire quantum dots, Applied Physics Letters **118**, 161107 (2021).

[3] E. Yeung, D. B. Northeast, J. Jin, P. Laferrière, M. Korkusinski, P. J. Poole, R. L. Williams, and D. Dalacu, On-chip indistinguishable photons using III-V nanowire/SiN hybrid integration, Physical Review B **108**, 195417 (2023).
